# Telephone Insanity

**Published:** 1890-09

**Authors:** 


### TELEPHONE INSANITY.

A tale is told by the Paris correspondent of the *London Daily Telegraph,* that may suggest Mark Twain's account of how Hank Morgan's sixth-century wife came to bestow the name of Hello Central on her first-born child. The Paris story is as follows:

A lady, about twenty-six years of age, employed in the chorus of one of the theatres, suddenly stopped in the middle of the rue des Petits-Carreaux and shouted at the top of her voice, "Hallo! hallo!" A crowd at once gathered around the young lady, who put her hands to-her mouth and ears in telephonic fashion. "Is that you, Saint Peter?" continued she, as if speaking into a tube. "Right, give me my keys? What? You can not be bothered? Then send your commissionaire. I must get home!" She repeated this several times, and at last the spectators came to the conclusion that she was wrong in her mind. A constable took her to the police station, where she went on in the same way, declaring that she heard distinctly through the telephone the celestial music of Paradise, that she could hear Saint Cecilia playing the piano, and that the chorus was composed of cherubim. She was sent into a hospital.—*New York Med. Journal.*

